# Effects of cushion box and closed let-down ladder usage on damage to corn during handling: physiological deterioration

**DOI:** 10.1186/s13007-022-00975-y

**Published:** 2022-12-22

**Authors:** Reza Shahbazi, Feizollah Shahbazi

**Affiliations:** Department of Biosystems Engineering, Lorestan University, Khoram Abbad, Iran

**Keywords:** Corn, Physiological damage, Handling, Drop height, Drop method

## Abstract

**Background:**

Corn seeds have a high susceptibility to mechanical damage due to their large size and mass. The main purpose of this study was to evaluate the effects of the cushion box and closed let-down ladder usage in minimizing the negative influence of the free fall on the storage potential of corn seeds. Corn seeds were evaluated for the extent of physiological damage by measuring the seed deterioration by the accelerated aging test (percentage loss in germination in the accelerated aging test), using three drop methods (free fall, with cushion box, and with closed let-down ladder) at three drop heights (5, 10, and 15 m) and five different moisture contents (10, 15, 20 and 25%).

**Results:**

The drop methods had a significant effect on the storage potential of corn seeds. Sample seeds dropped without a ladder (free fall) had a significantly higher average physiological quality loss of 13.87% (loss in accelerated aging germination). In the use of the cushion box, the average percentage loss in germination was calculated to be 11.38%, which was decreased by about 18% more than the free fall. Sample seeds dropped with the closed let-down ladder had a lower average percentage loss in the germination of 8.78%, which showed that the closed let-down ladder significantly helped to reduce mechanical damage to corn seeds by about 37% comparing free fall and by about 23% to the use of the cushion box. The amounts of loss in physiological quality of corn seeds increased significantly with increasing drop height and moisture content, but the use of the cushion box and closed let-down ladder systems somewhat reduced the adverse effect of the above factors. Empirical models were developed for the dependency of physiological damage to corn seeds due to the impact caused by free fall, on the drop height and moisture content at different drop methods.

**Conclusions:**

To minimize mechanical damage to seeds as they fall into the bin, a let-down ladder should be installed in the bin so that it can receive seeds from the filling spout with minimum damage.

## Background

From a technical point of view, seed processing, which includes threshing, cleaning, grading, handling and transportation, aims to increase the overall quality of the final seeds to meet the specific needs of the final consumers. Nevertheless, the reduction of the quality of the seeds during their processing seems inevitable. Mechanical damage caused by impact during the harvesting and processing of seeds, is one of the most important causes of quality reduction. This type of damage is a serious problem in the seed industry. Mechanical damage to seeds due to impact does not only depend on the processing operation but also depends on the impact velocity [1], seed structural characteristics [2, 3], seed variety [4], seed type [5], seed moisture [1, 6], ripening stage, level of fertilization [7, 8] and improper setting of the equipment [9]. Symptoms of mechanical damage to seeds may be in different forms [2, 3, 10]. External damage to seeds is easily visible, such as breakage and cracking of the seed. Internal damage included microscopic cracks and damage to the seed embryo. All these damages reduce the value of the product, reduce the shelf life of the product, create health problems, increase production and processing costs, reduce the efficiency of extracting nutrients from seeds and reduce the percentage germination of seeds [11, 12].

Mechanical damage reduces the shelf life of seeds by producing carbon dioxide gas and reducing dry weight [13–15]. Fractures, cracks and scratches on the seeds cause air and moisture to penetrate them and quick hydration of the living tissues, which reduces the ability to store and preserve the seeds [16].

Free fall of seeds during the handling and transportation is a critical stage in seed processing, in which seed damage occurs due to impact [17]. Depending on the type of operation, seeds may drop from a few meters in the field during unloading by a combine harvester and loading transporter truck, to a height of more than 50 m during unloading and loading in silos and export terminals. In these stages, the seeds fall from different heights and hit different surfaces as a result they are damaged. The damage caused in this case depends on various factors, The most important of which are the drop height, the conditions and properties of the seed (size, density, moisture content and temperature), ambient temperature and the type and angle of the contact surface [2, 18]. Using vertical pipes, in silos and another seed handling or processing systems allows the seeds to move at high speeds resulting in a high-intensity impact when seeds are discharged, which may result in mechanical damage to the seeds.

To reduce the mechanical damage to the seeds caused by free fall, the condition of the seeds and their falling conditions should be adjusted so that the severity of the impact should be reduced. Given that the conditions of the seeds are somewhat uncontrollable during the handling and free fall, the only factor that can be controlled or managed is falling conditions. As much as possible the drop height and speed of the falling seeds must be reduced to prevent the seeds from being hit on hard and winning surfaces. To protect the germination of seeds and their ability to maintain viability during storage, all points in all operations where seeds receive sharp blows or strike hard objects should be eliminated [19]. Any sharp blow to the seeds, either from the seeds falling and striking other seeds or a hard surface, or a moving object striking the seeds, can cause visible or internal seed damage. Most severe impacts between seed and hard surfaces during conditioning can be eliminated or reduced by minimizing drops and padding points of impact. A common method of feeding a separator installed some distance from the elevator leg is to increase the height of the elevator, to get a 45-degree (or more) slope in the discharge spout from the elevator to the bin. This often requires a long discharge spout, and seed velocity can build up to damaging levels as the seeds go from the elevator to the distant bin. This can be avoided by using a lower elevator combined with a vibrating or belt conveyor to bring seed horizontally to the elevator from the previous machine. Vibrating conveyors mounted on the floor, or trough-belt conveyors installed at elevator heads, beneath separators, or at other locations, can move seed horizontally and eliminate damaging drops. “Cushion boxes”, with rubber-padded baffles which slow down seed without causing a damaging impact, can be installed in seed pipes [20]. Let-down ladders which have rubber-padded baffle plates can let seeds down gently from a spout to a machine, elevator hopper, or bin location to reduce the drop height and speed of falling of the seeds when leaving the conveyors or in unloading or loading the seeds storage bins, stepping falling systems (ladder) should be used [20]. When seeds drop more than 1 m into a bin and strike other seeds or the bin wall, seed mechanical damage has been reported. Where possible, avoid long drops from elevator spouts to bins by placing the elevator closer to the bin and reducing the length of the downspout feeding the bin. To move the elevator closer to the bin, a horizontal conveyor (usually a self-cleaning vibrating conveyor) can be used to bring seed from the previous machine to the elevator feed hopper. Where long down-spout drops cannot be eliminated and seeds build up damaging velocity in falling a long spout, or drop a long distance from the spout into the bin, several methods can decrease the force of the impact when seeds fall into the bin. Inside the bin, rubber-padded “seed step-down ladders or baffles” or rubber-padded spiral chutes, can carry seed safely from the spout down into the bin. They have open sides, and automatically discharge seeds at a higher point as the seed level in the bin rises. Spiral slide let-downs can also be installed in bins. One of these systems should be installed inside every bin To reduce the drop height and speed of falling of the grains when leaving the conveyors or in unloading or loading the grains storage bins, stepping falling systems (ladder) should be used [20, 21]. Impact damage to seeds has been the subject of much research due to reduced crop quality during harvesting, handling and processing [22–28]. Various drop tests studied were implemented with the seeds of some agricultural products such as chickpeas [17], corn [21, 29–31], soybean [19, 30] and wheat [30]. The results of the above research showed that the mechanical damage to the seeds due to the impact caused by free fall increased with drop height.

Fiscus et al. [30] reported that the damage to grains caused by falling more than 13 m, was higher than that caused by using either a grain thrower or a bucket elevator. When tested under the same conditions, the impact damage due to free fall depended on the type of grain. Wheat was less susceptible to damage than soybeans (percentage damage was less than 0.4%) and soybeans were less susceptible to impact damage than corn [32]. Bergen et al. [33] in a study on damage to ‘Trapper’ peas and ‘Laird’ lentils in free fall, observed that seeds dropped from a greater height caused more seed damage on all three selected surfaces, namely steel, plywood, and concrete. Seeds with lower moisture content reportedly incurred more damage. Perry and Hall [34] evaluated the mechanical damage to pea beans using drop tests. Damage of pea beans was found: (a) to vary proportionately with drop height (3.4, 6.9, and 13.7 m); (b) to be reduced by one-half or more when temperatures were increased from −4 °C to the 13 and 21 °C range; and, (c) to increase at 14% moisture and particularly at 12% as compared to 16 and 18% moisture. Tang et al. [35], in a study of the breakage susceptibility of lentil seeds, observed that for the same moisture content, the breakage in samples was higher at −25 °C than at room temperature of 22 °C. The increase in breakage at −25 °C was attributed to the crystallization of available free water. Nevertheless, when the moisture content was less than 10%, the lower temperature did not cause an increase in breakage. In researches by Shahbazi and Shahbazi [21] studied the effects of cushion box and closed let-down usage in reducing the mechanical damage of corn seeds due to free fall at different drop heights and moisture contents and reported that the use of those systems reduced the amount of damage to corn seeds in the forms of breakage percentage and cracking index.

Corn seeds are non-chaffy and because of their large size and mass are susceptible to mechanical damage during processing. This predisposed the seeds to physical and physiological deterioration and ultimately loss of seed quality [27, 36]. In addition, corn seeds are very fragile due to the lack of gluten, which is a natural internal binder in the grains [32].

However, information relating to the physiological deterioration of corn seeds to mechanical damage due to free fall during processing is limited. Therefore, the objectives of this study were (1) to qualify and quantify the amount of physiological quality loss (loss in accelerated aging test germination) of corn seeds during handling caused by free fall; (2) to evaluate the effects of the cushion box and closed let-down ladder usage in minimizing physiological damage to corn seeds during free fall, related to moisture content and drop height.

## Materials and methods

In this study, the effects of the drop method, drop height and seed moisture on the physiological damage to corn seed caused by the impact due to free fall were invested. Corn seeds of KSC 705, which were harvested manually at the ripening stage, were used for impact tests. Seeds were stored at 5 °C temperate and about 90% humidity, until the start time of the experiments. Before the free fall impact tests, the initial moisture content and germination of seed samples were determined. Moisture was determined based on the ASABE standard S352.2 [37]. The initial moisture of the seeds was about 10%. Seeds with higher levels of moisture (15, 20 and 25%) were prepared by adding special pre-calculated distill water on samples and then kept in closed plastic envelopes at a temperature of 4 °C for 10 days. The initial germination of the seeds was about 97%.

**Fig. 1:**
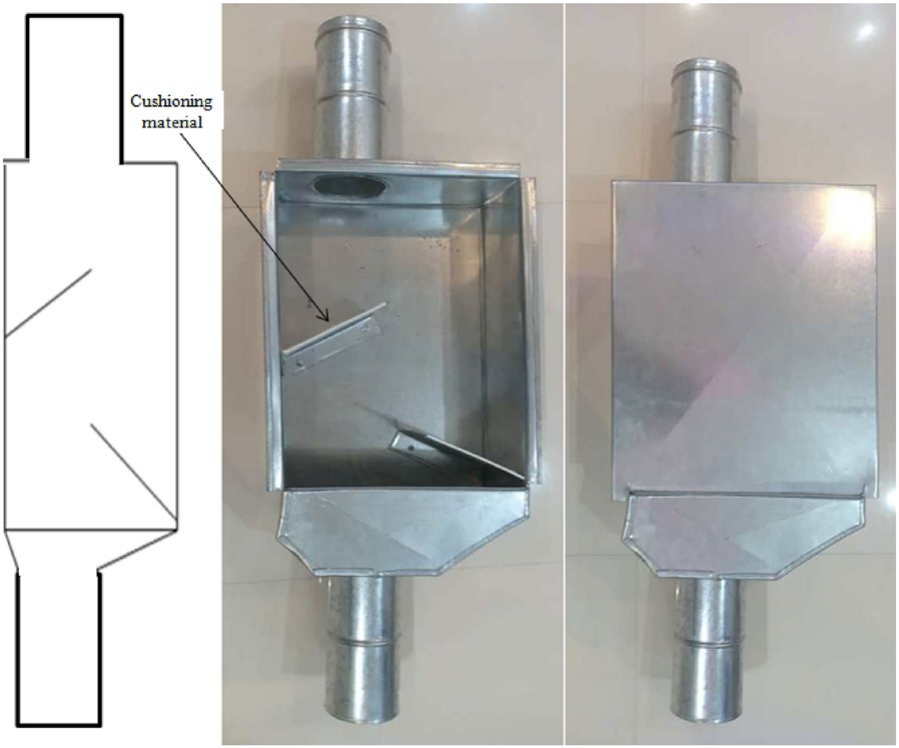
Schematic and the sample of the cushion box used in research

**Fig. 2:**
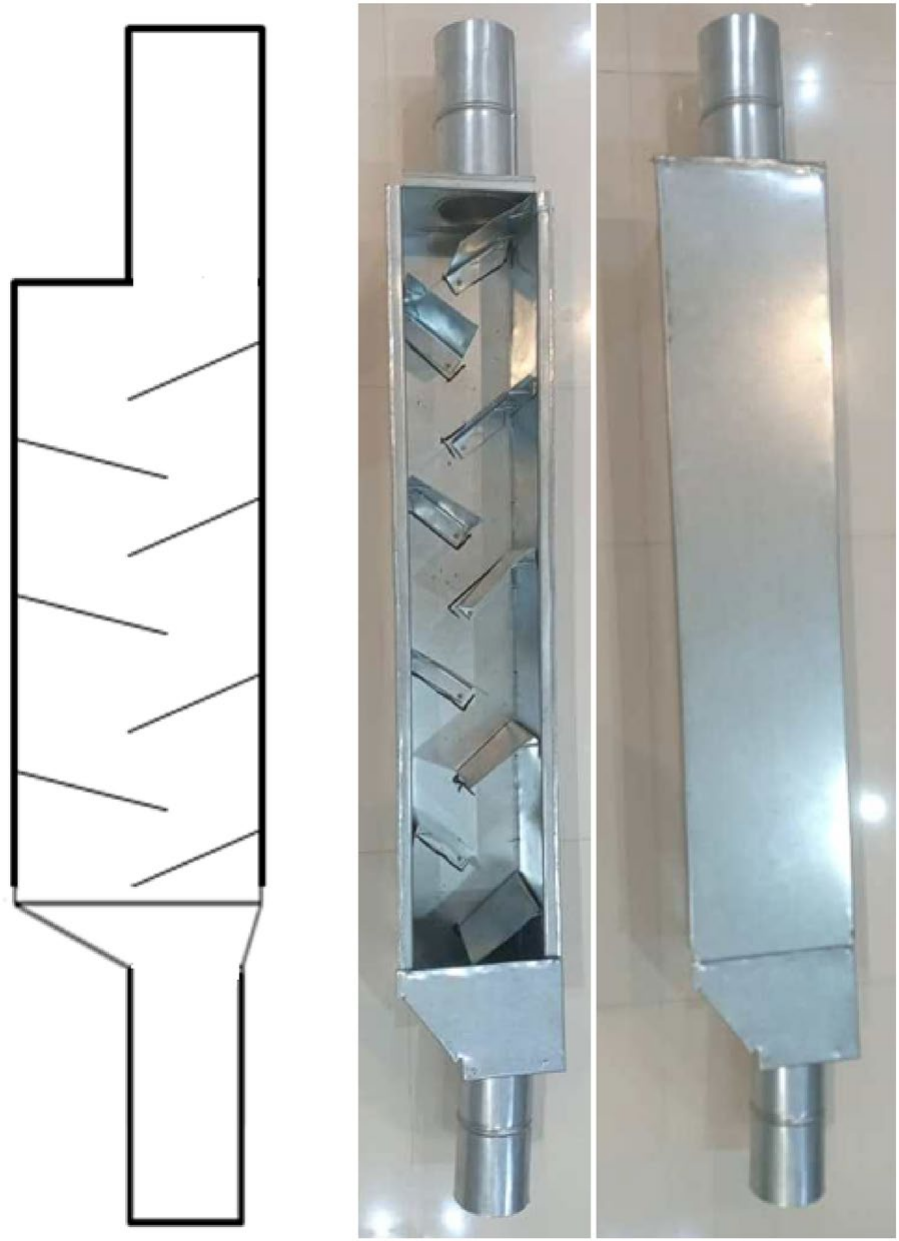
Schematic and the sample of the closed let-down ladder

Laboratory tests were used to simulate free fall and evaluate the effect of the cushion box and closed let-down ladder usage in minimizing physiological damage to corn seeds. Two dropping systems (cushion box and closed let-down ladder) were designed and developed. Figure 1 shows the schematic and the sample of the cushion box used in this research. This system consists of a box 100 cm in length and 20 cm in width [twice the width of the transfer pipe diameter (10 cm)]. Inside the box, there are two sloped blades 22 cm in length, installed at 45° in the opposite directions of seed flow and cushioned with rubber with a thickness of 4 mm [21]. Based on the grain transfer pipe length, the cushion box can be installed at different distances along the pipe to minimize mechanical injury caused by impact. The box was made of galvanized steel with a thickness of 7 mm. Figure 2 shows the closed let-down ladder which has sloped rubber-padded baffle plates. This type of system lets seeds fall gently to the bin without causing a mechanical impact on the seeds. The structure of this system consists of a cubic box that is 100 cm long and 15 cm in width [1.5 times the width of the transfer pipe diameter (10 cm)] The ladder was made of a galvanized still sheet with a thickness of 7 mm [21]. As shown in Figs. 1 and 2, the dimensions considered for the cushion box or the closed let-down ladder, the space for the flow of grains is equal to the space of a pipe without a system, which may cause the throughput to be equal to a pipe without a system. Therefore, there are not any negative effects when using the cushion box or the closed let-down ladder on the rate and time of corn transshipment in commercial operation.

**Fig. 3:**
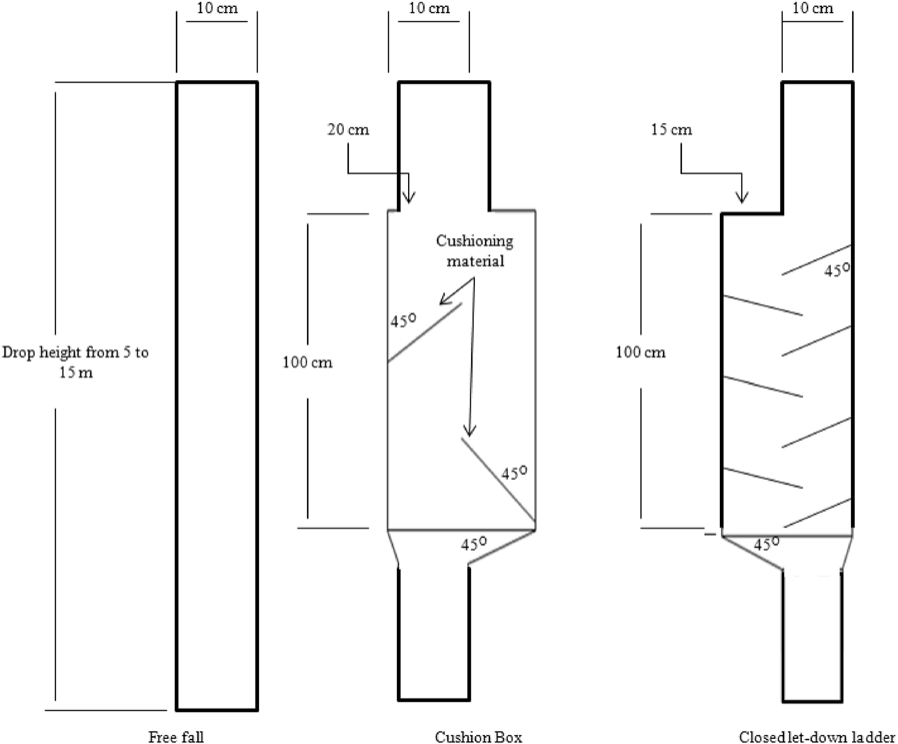
Schematic of three systems used for drop tests

Cron seed samples with moisture contents of 10, 15, 20 and 25 which are typical moisture contents at harvest and postharvest operations of corn, were dropped using three different methods: (1) with a cushion box, (2) with a closed let-down ladder, and (3) with free fall using PVC pipe (with a diameter of 10 cm) without a device (Fig. 3).

Three drop heights of 5, 10, and 15 m were selected. These drop heights are typically indicative of a situation on the farm, at a grain cleaning plant, or in a grain elevator. For obtaining different drop heights, PVC pipes of 10 cm diameter were set up. A hopper with a 40 mm diameter opening was attached to the top of the pipe. The dropping rate of kernel samples was regulated using a gate attached at the bottom of the hopper. The flow rate of kernel samples was regulated at 0.25 kg/s. For drop tests, 5 kg of pre-sieved seed samples (whole seeds without external damage) were placed in the hopper and then dropped into the desired system. To prevent the scattering of the grains at the end of the systems, a wooden chamber was placed at floor level. The concrete floor of the chamber was installed at an inclination of 45° to simulate a drop in an empty bottom bin. After drop tests, the samples were then collected from the chamber and transferred to plastic bags for damage analysis.

### Damage assessment

After free fall tests, the seed samples were graded manually. The seeds of each sample treatment were divided as externally or physically damaged and undamaged by visual inspection using a magnifying lens [38]. To determine the damage to seeds due to free all as affected by the drop method, drop height and moisture content, the method for measuring the deterioration of seeds by the accelerated aging test was used. The undamaged seeds in each sample lot were subjected to the accelerated aging test to determine the storability and check for internal damage to the seed due to free fall. To perform the accelerated aging test, 50 g of corn seeds from different samples (control samples and free fall tested samples) were weighed and placed in an accelerated aging chamber at 42 °C and relative humidity of 100%, for 72 h, and then the accelerated aging germination percentage was calculated [39]. The percentage loss in the accelerated aging test germination was calculated based on the difference between the germination percentage of the control samples (untreated) and the tested samples.

### Measurement of corn seeds velocity

A video camera was used to record the velocities (single seeds and mass flow) of seeds just before they hit the test surface in three drop methods. A distance of approximately 1 m was maintained between the lower end of the PVC pipe and the test surface so that velocities of seeds coming out of the pipe could be recorded. A set of horizontal lines were drawn at an interval of 5 cm and placed in the background for ease in observing the distance traveled by seeds. Images of both stream and individual seeds were taken to observe if air resistance affected their speeds. Because tracking seeds in mass flow was difficult, the velocities of seeds at the beginning and the end of mass flow were recorded.

### Statistical analysis

The factorial experiment was conducted as a randomized design. The main effects were the dropping method (free fall, cushion box and closed let-down ladder), moisture content (10, 15, 20 and 25), and drop height (5, 10, and 15 m). There were three replications for measurements of the percentage loss in the accelerated aging test germination (the dependent variable). Thus, there were 108 (3 × 4 × 3 × 3) observations. The main treatments and their interactions were analyzed using analysis of variance (ANOVA) by using SPSS software (version 19). For graphs and tables, Microsoft Excel was used. The level of significance was shown as *p < 0.05 and **p < 0.01 by applying Duncan’s multiple range tests.

## Results and discussion

In this study, physiological deterioration (damage) to KSC 705 corn hybrid due to free fall and the effects of the drop method, drop height, and moisture content were investigated. The results of the study illustrated that the physiological deterioration (percentage loss in the accelerated aging test germination) of corn seeds was affected by the drop method, drop height and moisture content. Table 1 shows the results of the analysis of variance for the percentage of physiological damage to corn seeds in different treatments of drop method, drop height, and moisture content. The drop method, drop height, and moisture content appeared to have significant effects on the percentage of physiological damage to corn seeds (*p* < 0.01). Furthermore, the interaction between drop method × drop height, drop method × moisture content and drop height × moisture content and the interaction between three independent variables had significant effects (*p* < 0.01) on the percentage loss in the accelerated aging test germination of corn seeds.

**Table 1:** Analyses of variance for the percentage loss in the accelerated aging test germination of corn seeds as affected by drop method, drop height and moisture content

Source	*df*	Sum of squares	Mean square	F
Drop method (DM)	2	467.82	233.91	653.97**
Drop height (DH)	2	971.31	485.66	1357.81**
DM × DH	4	7.06	1.76	4.93*
Moisture content (MC)	3	805.99	268.66	751.14**
DM × MC	6	9.20	1.53	4.28*
DH × MC	6	10.06	1.68	4.69**
DM × DH × MC	12	20.12	1.68	4.69**
Error	72	25.75	0.36	
Total	10	16,210.18		

**p < 0.01, *p < 0.05

Table 2 shows the results of means comparison for the percentage physiological damage (percentage loss in the accelerated aging test germination) to corn seeds used in drop tests. All three independent variables, namely, drop methods, drop heights, and moisture content, had significant effects (*p* = 0.05) on the measured values. The percentage of physiological damage to corn seeds significantly decreased by using the cushion box and closed let-down ladder. In addition, the percent physiological damage to seeds increased as the drop height or the moisture of seeds increased within the tested ranges.

**Table 2:** Duncan’s multiple range tests compare the means of the percentage loss in the accelerated aging test germination percentage of corn seed at different treatments of drop method, drop height, and moisture content

Treatment	Loss in the accelerated aging test germination (%)
*Drop method*	
Free fall	13.87 a
Cushion box	11.38 b
Closed let-down ladder	8.78 c
*Drop height (m)*	
5	8.53 c
10	10.70 b
15	13.26 a
*Moisture content (%)*	
10	7.83 d
15	9.95 c
20	12.47 b
25	15.12 a

a–d: Mean values in the columns with the same letter are not significantly different (p < 0.05)

From the data in Table 2, it is evident that there were significant differences between the average percentage of physiological damage to the seeds using the different drop methods (*p* < 0.05). In different test conditions, which included different levels of seeds’ moisture content and drop height, sample seeds that dropped using a pipe without a system (free fall) had a significantly higher average percentage loss in the accelerated aging test germination of 13.87%. In the case of using the cushion box, the means of the percentage loss in germination was 11.38%, which was approximately 18% lower than that of free fall. Samples seeds were dropped using the closed let-down ladder had a significantly lower average percentage loss in the accelerated aging test germination of 8.78%, showing that the closed let-down ladder significantly helped to reduce physiological damage to the corn seeds by approximately 37% compared to that of free fall and approximately 23% compared to that of the cushion box, which effectively prevented physiological deterioration of the seeds and reduced the negative influence on the storage potential of corn seeds.

**Fig. 4:**
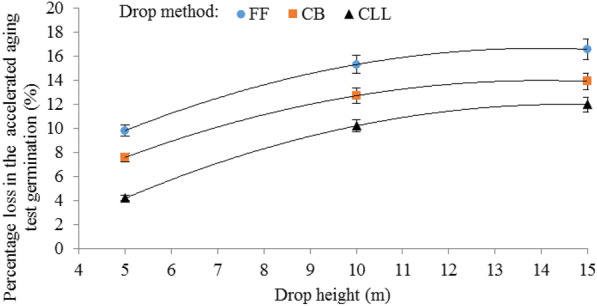
Interaction effect of drop method and drop height on the percentage loss in the accelerated aging germination of corn seeds. *FF* fre fall, *CB* cushion box and *CLL* closed let-down ladder

This may be due to the differences between the velocities of the seeds that were dropped using the three different drop methods. Table 3 shows the average velocities (single kernel and mass flow) for corn seeds dropped from various heights using the three different drop methods. In the free fall drop method, the average velocities (single seed and mass flow) measured were as expected much higher compared to dropping the seeds using either the cushion box or the closed let-down ladder. Also, seeds dropped using the closed let-down ladder had lower velocity values compared to the cushion box at all three drop heights. Corn seed damage increased as the impact velocity increased which was similar to the cases for chickpeas [1], soybeans [40] and wheat [41]. In free fall the average mass flow velocities of seeds when dropped from the heights of 5, 10, and 15 m were 7.85, 10.12, and 13.90 m/s, respectively. At these velocities, the average percentage losses in the germination of corn seeds were 9.79%, 15.28%, and 16.55%, respectively (Fig. 4). The average mass flow velocities of seeds when dropped using the closed let-down ladder from the heights of 5, 10, and 15 m were 3.65, 5.03, and 7.69 m/s, respectively, at these velocities, the means of the percentage losses in the germination of corn seeds were 4.18%, 10.19%, and 11.96%, respectively (Fig. 4), showing that the lower mass flow velocity (rate) of seeds during the falling was achieved as a result of the application of the closed let-down ladder (Table 3), resulting in a slower slide of the seeds and thereby, limiting the proportion of seeds damaged in the test comparing free fall. Therefore, the innovative system for storing corn seeds allows for its full use in practice. This improves the quality of corn seeds and allows them to be safely stored in silos. In addition, in different drop methods, mass flow velocity measurement, velocities were higher compared to dropping the seeds individually. Seeds dropped individually had lower velocity values which may be due to the effect of air resistance encountered in the drop tubes.

**Table 3:** Average velocities for corn seeds dropped from various heights in three drop methods

Drop height (m)	Drop method					
	Free fall		Cushion box		Closed let-down ladder	
	Velocity (single kernel) (m/s)	Velocity (mass flow) (m/s)	Velocity (single kernel) (m/s)	Velocity (mass flow) (m/s)	Velocity (single kernel) (m/s)	Velocity (mass flow) (m/s)
5	7.25	7.85	4.85	5.32	3.20	3.65
10	9.45	10.12	7.36	8.45	4.65	5.03
15	11.02	13.90	9.89	11.13	6.32	7.69

No published results exist in the literature showing these same results utilizing cushion box and closed let-down ladder usage in minimizing physiological damage (seed deterioration) to corn seeds during free fall. The effectiveness of cushion box, spout retarders and retro-air retarders in reducing damage to corn were studied by Stephens and Foster [42]. It appeared that the flow decelerators were able to reduce handling damage, however, the degree of reduction was small. Shah et al. [19] reported that bins filled with soybeans using a bean ladder had a lower percentage of damaged or cracked beans and exhibited a higher quality, including germination levels, than seeds in the bin filled without a ladder. In a study on the improvement of grain quality by Bartkowiak et al. [32] they reported that corn seeds with a moisture content of about 18% dropped using a cascade chute from 6 m height showed four and five times less damage compared with the free fall. They also reported that a 6 m cascade chute considerably decreased the velocity of the filling grains when loading the silo. In a study on the effects of cushion box and closed let-down ladder usage in reducing the mechanical damage of corn seeds due to free fall by Shahbazi and Shahbazi [21] they reported that at different drop heights and moisture contents and reported that, sample corn seeds that dropped without a ladder (free fall) had higher internal damage in the form cracks index of 144.8 in comparison to 103.3 and 51.6 in use of the cushion box and closed let-down ladder respectively, which showed that the closed let-down ladder significantly helped to reduce mechanical damage to corn seeds by about 64% comparison free fall and about 50% to the use of the cushion box.

There were differences (*p* < 0.05) between the physiological damage to seeds when using the cushion box compared to that of the closed let-down ladder (Table 2). This could be caused by differences in the design and operation of the above systems. In the cushion box, because of the position of its blades, which are in the opposite direction of the seeds' flow, an additional impact may be created, which could result in more damage to the seeds when compared to the closed let-down ladder. Additionally, this difference may be caused by differences in the corn kernel's acceleration and average mass flow velocities during dropping using the cushion box and the closed letdown ladder from different heights (Table 3). Therefore, further research is needed to investigate this issue.

The percentage of physiological damage to corn seeds increased significantly with increasing drop height (Table 2). Furthermore, there were significant differences between the average percentage losses in the accelerated aging germination of corn seeds at different levels of drop heights (*p* < 0.05). In different experimental conditions, including different drop methods and different levels of moisture content, the least damage to seeds (7.18% loss in the accelerated aging test germination) was caused at a drop height of 5 m. By increasing the drop height from 5 to 10 m, the mean values of the percentage loss in the accelerated aging test germination of seeds increased by 1.79 times and increased from 7.18 to 12.72%. At a drop height of 15 m, there was higher physiological damage to corn seeds and the percentage loss in the accelerated aging test germination of seeds at this height was equal to 14.13%, which increased by about 2 times compared to the drop height of 5 m, and by about 1.2 times compared to the drop height of 10 m. According to the law of conservation of energy *E* = *mgh*, where *E* denotes impact energy (J), *m* denotes kernel or sample mass (kg), *g* denotes acceleration of gravity (9.81 m/s^2^) and *h* denotes drop height (m), it is predictable that with increasing drop height, the amount of applied impact energy to the seeds will be increased, and, as a result, the amount of damage will increase. Also, this is because of the increasing kernel velocity with drop height (Table 3), which results in a large impact force [30]. According to data in Table 3, at different drop methods, the mass velocity of corn seeds increased significantly with increasing drop height increasing the percentage loss in the germination of corn seeds. Foster and Holman [43] reported that when the drop height was more than 15 m, the velocity of the grain stream could exceed the single kernel velocity because, when the grain stream was dropped as a whole, the drag forces applied to the individual grains were not all the same. They suggested limiting the drop height to 12 m to reduce free fall damage. Therefore, it is necessary to reduce the drop height of the grains/seeds as much as possible. One of the ways to do this is the use of ladders systems that were mentioned in the previous section. The adverse effect of increasing drop height was similar to what was reported by Bergen et al. [33] on ‘Laird’ lentils. Perry and Hall [34] evaluated the mechanical damage to pea beans using drop tests and observed that the damage to pea beans was found to vary proportionately with drop height. Furthermore, similar results were reported by Fiscus et al. [30] about mechanical damage to corn, soybeans and beans. Gatongi [44] reported that mechanical damage during corn grain processing was affected by drop height and moisture content. Asiedu [45] reported a sharp decrease in the germination percentage of corn seeds with increasing drop height on hard surfaces.

The increase in moisture resulted in an increased percentage of physiological damage to corn seeds due to free fall (Table 2). As the moisture content of seeds increased from 10 to 25%, the percentage loss in the accelerated aging germination of corn seeds increased from 7.83 to 15.12%. At higher moisture contents seeds are more elastic, which may cause the transfer of the energy of the impact to the internal parts of the seed, resulting in damage to the embryo, therefore, they are more susceptible to physiological damage during free fall and other processes, similarly reported by Khazaei et al. [41] for wheat and Shahbazi et al. [38] for Pinto beans.

Figure 4 shows the interaction effect of the drop method and drop height on the percentage of physiological damage to corn seeds. The effect of drop height was highly critical when seeds were dropped using a free fall or cushion box. This difference was higher at the drop height of 15 m compared with the 10 m and 5 m heights. The same trend was observed using the closed let-down ladder, in which the damage was greater at the drop height of 15 m, but the difference in seeds damage at the three drop heights when seeds were dropped using the closed let-down ladder was significantly lower. In Fig. 4, the lowest percentage loss in the accelerated aging test germination of corn seeds was 4.18%, which was created by the interaction effect of the closed let-down ladder and the drop height of 5 m. The highest percentage loss in the accelerated aging germination of corn seeds was 16.55%, created in the case of free fall and drop height of 15 m. It is evident that, in all drop heights, the lower damage was caused by the use of the closed let-down ladder and the highest amount was caused by the free fall. Furthermore, in different drop heights, a medium amount of damage was related to the use of the cushion box. The dependency of percentage physiological damage to corn seeds (*PPD*, %) on drop height (*DH*, m) was expressed by the following bets-fit equations for drop methods of free fall, cushion box and closed let-down ladder respectively:

1 $$PPD = - 0.08DH^{2} + 2.36DH + 0.09\,R^{2} = 0.99\,\quad {\text{at}}\,{\text{free}}\,{\text{fall}}$$

2 $$PPD = - 0.08DH^{2} + 2.21DH - 1.49\,R^{2} = 0.99\,\quad {\text{at}}\,{\text{cushion}}\,{\text{box}}$$

3 $$PPD = - 0.08DH^{2} + 2.47DH - 6.07\,R^{2} = 0.99\,\quad {\text{at}}\,{\text{closed}}\,{\text{let - down}}\,{\text{ladder}}$$

The regression statistics for the models indicated that all the indexes (terms) were significant at the level of 99.99% on the accuracy of the models. As follows from the relations (1)–(3) the effect of drop height (*DH*) is stronger on the percentage physiological damage to corn seeds (*PPD*) at the drop method of free fall (higher indexes at free fall) than cushion box and closed let-down ladder, showing that the use of the cushion box and closed let-down ladder systems somewhat reduced the adverse effect of the drop height.

**Fig. 5:**
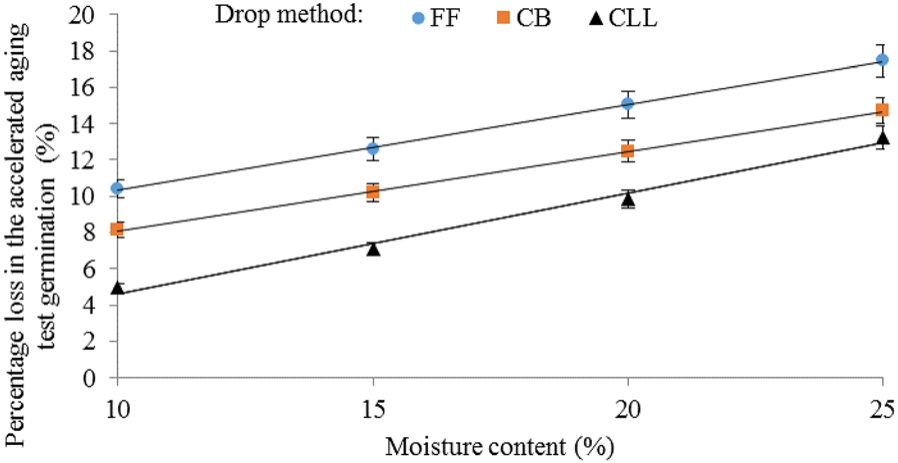
Interaction effects of drop method and moisture content on the percentage loss in the accelerated aging test germination of corn seeds. *FF* fre fall, *CB* cushion box, *CLL* closed let-down ladder

Shown in Fig. 5 is the interaction effect of the drop method and moisture content on the percentage loss in the accelerated aging test germination of corn seeds. In all the drop methods, as the moisture level increased, the damage to seeds increased. The difference in seed damage at the four moisture levels when seeds were dropped using the closed let-down ladder was significantly lower than the seeds that dropped using free fall. The effect of the moisture level was less critical when seeds were dropped using the closed let-down ladder. The lower damage was 4.93%, which was created by the interaction of the use of the closed let-down ladder and moisture content of 10%. The highest damage was 17.44%, which was created in the case of free fall with a moisture content of 25%. In all seed moisture levels, the medium damage was related to the use of the cushion box. The dependency of percentage physiological damage to corn seeds (*PPD*, %) on moisture content (*MC*, %) was expressed by the following bets-fit equations for drop methods of free fall, cushion box and closed let-down ladder respectively:

4 $$PPD = 0.47MC + 5.63\,R^{2} = 0.999\,\quad {\text{at}}\,{\text{free}}\,{\text{fall}}$$

5 $$PPD = 0.44MC^{2} + 3.68\,R^{2} = 0.999\,\quad {\text{at}}\,{\text{cushion}}\,{\text{box}}$$

6 $$PPD = 0.55MC - 0.90\,R^{2} = 0.999\quad \,{\text{at}}\,{\text{closed}}\,{\text{let - down}}\,{\text{ladder}}$$

The regression statistics for the models indicated that all the indexes (terms) were significant at the level of 99.99% on the accuracy of the models. As follows from the relations (4)–(6) the effect of moisture content (*MC*) is stronger on the percentage physiological damage to corn seeds (*PPD*, %) at the drop method of free fall (higher indexes at free fall) than cushion box and closed let-down ladder, showing that the use of the cushion box and closed let-down ladder systems somewhat reduced the adverse effect of the moisture content.

**Fig. 6:**
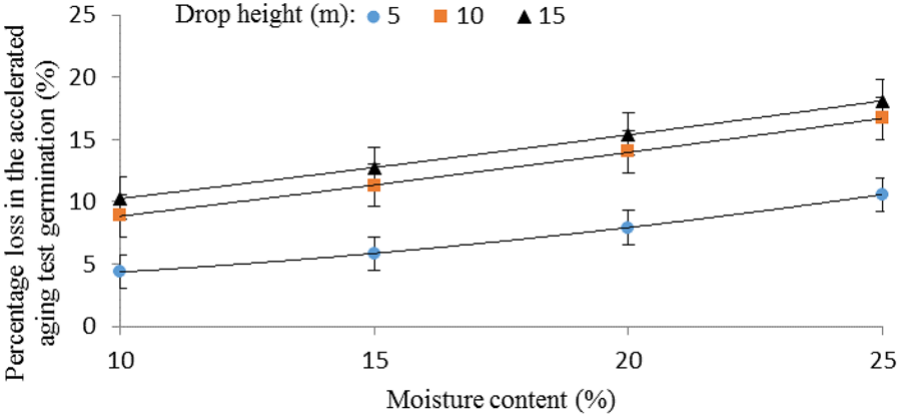
Interaction effects of drop height and moisture content on the percentage loss in the accelerated aging germination of corn seeds

Figure 6 shows the interaction of drop height and moisture content on the percentage loss in the accelerated aging test germination of corn seeds. As the moisture level decreased, the physiological deterioration of seeds increased at a higher rate with the increase in drop height.

The means of the percentage loss in the accelerated aging test germination of corn seeds at various drop methods, drop heights and moisture contents (interaction between three independent variables) are shown in Table 4. From the data for the average percentage loss in the accelerated aging germination of corn seeds after the drop tests under various conditions in Table 4, it is observed that the percentage loss in the germination of seeds was affected by the drop height, impact surface and moisture content. In all drop methods, increased drop height and increased moisture content caused increased trends in the mean values of physiological damage to seeds due to free fall. Corn seeds dropped from the height of 15 m, averaging a percentage loss in the germination of 13.08% on free fall, 10.64% on cushion box and 7.10% on closed let-down ladder, at the moisture content of 10%. While at the moisture content of 25% and the same drop height of 15 m, the percentage loss in the germination of seeds was high as 20.12% on free fall, 17.20% on cushion box and 16.97% on closed let-down ladder.

**Table 4:** Percentage loss in the accelerated aging test germination of corn seeds when dropped as affected by the drop height, moisture content and drop method

Drop height (m)	Moisture content (%)	Drop method		
		Free fall	Cushion box	Closed let-down ladder
5	10	6.33	4.33	2.37
	15	8.51	6.37	2.69
	20	10.97	8.67	4.14
	25	13.36	10.90	7.50
10	10	11.82	9.46	5.33
	15	14.00	11.49	8.39
	20	16.46	13.79	11.84
	25	18.85	16.02	15.19
15	10	13.08	10.64	7.10
	15	15.26	12.67	10.16
	20	17.72	14.97	13.62
	25	20.12	17.20	16.97

## Conclusions

In this study, the effects of three drop methods in corn seed handling (related to moisture content and drop height) on corn seed's physiological deterioration by the accelerated aging test (percentage loss in germination in the accelerated aging test) were compared. From the results obtained, it was observed that: at different levels of moisture contents and drop heights, the use of the cushion box and closed let-down ladder effectively and significantly minimized physiological damage to corn seeds during free fall. In addition, there was a significant difference between the effects of the cushion box and the closed let-down ladder. In the use of cushion box, the physiological damage to the seeds was about 18% less than the pipe without a system. In the use of the closed let-down ladder, the physiological damage of corn seeds was reduced by about 437%, in comparison with the pipe without a ladder (free fall), and was about 23% lower compared to the use of the closed let-down ladder, which effectively prevented physiological damage to seeds and reduced physiological deterioration. In addition, the lower mass flow velocity (rate) of seeds during the falling was achieved as a result of the application of the closed let-down ladder, resulting in a slower slide of the seeds and thereby, limiting the proportion of seeds damaged in the test. Therefore, the innovative system for storing corn seeds allows for its full use in practice. This improves the storability quality of corn seeds and allows them to be safely stored in silos. Drop height was a critical factor. Increased drop height caused increased physiological deterioration of seeds. The least physiological damage to seeds was caused at drop heights of 5 and 10 m. At the drop height of 15 m, higher deterioration of corn seeds has been caused and the percentage loss in germination in the accelerated aging test at this height was equal to 13.26%, which was increased by about 36% compared to the drop height of 5 m, and about 19% compared to the drop height of 10 m. As the moisture level increased, the physiological deterioration of corn seeds due to free fall increased. Seeds with 25% moisture content had a higher mean percentage loss in germination in the accelerated aging test of 15.12%, compared to 12.47%, 9.95%, and 7.83% at moisture contents of 20, 15, and 10%, respectively. Therefore, the recommendations of this study for the design of the devices in corn seed harvest and postharvest operations for preventing mechanical damage caused by free fall regarding the condition of the grains and their falling conditions should be adjusted so that the severity of the impact is reduced. For these purposes, drop height should be minimized. In addition, to minimize mechanical damage to seeds as they fall into the bin, a let-down ladder should be installed in the bin so that it can receive seeds from the filling spout with minimum damage.

## Data Availability

Not applicable.

## References

[1] Shahbazi F (2011). Impact damage to chickpea seeds as affected by moisture content and impact velocity. Appl Eng Agric.

[2] Chen Z, Wassgren C, Ambrose RK (2021). Measured damage resistance of corn and wheat kernels to compression, friction, and repeated impacts. Powder Technol.

[3] Shahbazi F (2021). Mechanical Damage to Agricultural Grains (Causes and Solutions).

[4] Shahbazi F (2012). A study on the seed susceptibility of wheat (*Triticum aestivum* L.) cultivars to impact damage. J Agric Sci Technol.

[5] Shahbazi F, Valizade S, Dowlatshah A (2017). Mechanical damage to green and red lentil seeds. Food Sci Nutr.

[6] Shahbazi F, Saffar A, Analooei M (2011). Mechanical damage to navy beans as affected by moisture content, impact velocity and seed orientation. Qual Assur Saf Crops Foods.

[7] Shahbazi F, Sharafi R, Moomevandi SJ, Daneshvar M (2015). Influence of foliar iron fertilization rate on the breakage susceptibility of wheat seeds. J Plant Nutr.

[8] Shahbazi F, Sharafi R, Moomevandi SJ, Daneshvar M (2015). Mechanical damage to wheat seeds as affected by phosphorus and iron fertilization rate. Qual Assur Saf Crops Foods.

[9] Shahbazi F, Valizadeh S, Dolatshaie A (2012). Correlating the data on the mechanical damage to mung bean seeds under impact loading. Int J Food Eng.

[10] Paulsen MR, Singh M, Singh V. Measurement and maintenance of corn quality. In: Corn. AACC International Press; 2019. p. 165–211.

[11] Liu S, Song F, Liu F, Zhu X, Xu H (2012). Effect of planting density on root lodging resistance and its relationship to nodal root growth characteristics in maize (*Zea mays* L.). J Agric Sci.

[12] Meyers TP, Hollinger SE (2000). An assessment of storage terms in the surface energy balance of maize and soybean. Agric For Meteorol.

[13] Fan Y, Jacob KV, Freireich B, Lueptow RM (2017). Segregation of granular materials in bounded heap flow: a review. Powder Technol.

[14] Deng T, Garg V, Salehi H, Bradley MS (2021). Correlations between segregation intensity and material properties such as particle sizes and adhesions and novel methods for assessment. Powder Technol.

[15] Narendran RB, Jian F, Jayas DS, Fields PG, White ND (2019). Segregation of canola, kidney bean, and soybean in wheat bulks during bin loading. Powder Technol.

[16] Da Silva AB, Scatolini TB, Danao MG, Gates RS, Rausch KD. Effects of splits content on dry matter loss rates of soybeans measured using a static grain respiration measurement system. In: 2018 ASABE Annual International Meeting; 2018. p. 1.

[17] Delfan F, Shahbazi F, Esvand HR. Impact damage to chickpea seeds during free fall. In: International Agrophysics (INTAGRO-00060-2022-03) (Accepted paper); 2022.

[18] Chen Z, Wassgren C, Ambrose K (2020). A review of grain kernel damage: mechanisms, modeling, and testing procedures. Trans ASABE.

[19] Shah FS, Watson CE, Meredith ND, Bohn PA, Martin B (2001). Effect of bean ladder usage on mechanical damage during soybean seed conditioning. Seed Technol.

[20] Gregg B, Billups G (2016). Seed conditioning: technology—parts A & B.

[21] Shahbazi R, Shahbazi F (2022). Effects of cushion box and closed let-down ladder usage on mechanical damage during corn kernel handling: cracking. J Stored Prod Res.

[22] Su Y, Cui T, Zhang D, Xia G, Gao X, He X, Xu Y (2019). MLR and experimental testing for characterization and classification of damage resistance of maize hybrids based on mechanical properties. J Food Process Eng.

[23] Su Y, Cui T, Zhang D, Xia G, Gao X, He X, Xu Y (2019). Damage resistance and compressive properties of bulk maize kernels at varying pressing factors: experiments and modeling. J Food Process Eng.

[24] Su Y, Cui T, Zhang D, Xia G, Gao X, He X, Xu Y (2020). Effects of shape feature on compression characteristics and crack rules of maize kernel. J Food Process Preserv.

[25] Su Y, Xu Y, Cui T, Gao X, Xia G, Li Y, Qiao M, Yu Y (2021). HANDY: a device for assessing resistance to mechanical crushing of maize kernel. Plant Methods.

[26] Gu RL, Huang R, Jia GY, Yuan ZP, Ren LS, Li LI, Wang JH (2019). Effect of mechanical threshing on damage and vigor of maize seed threshed at different moisture contents. J Integr Agric.

[27] Guo D. Kernel and bulk density changes due to moisture content, mechanical damage, and insect damage, Doctoral dissertation. Purdue University.

[28] Kim TH, Hampton JG, Opara LU, Hardacre AK, Mackay BR (2002). Effects of maize grain size, shape and hardness on drying rate and the occurrence of stress cracks. J Sci Food Agric.

[29] Li X, Ma F, Gao L (2009). Dropping impact experiment on corn seeds. Trans Chin Soc Agric Eng.

[30] Fiscus DE, Foster GH, Kaufmami HH (1971). Physical damage of grain caused by various handling techniques. Trans ASAE.

[31] Foster GH. Grain breakage caused by commercial handling methods. Agricultural Research Service, United States Department of Agriculture; 1973.

[32] Bartkowiak A, Gracz W, Marcinkowski D, Skrzypek D, Wojtaszek S (2019). Research on quality of maize grain as a result of the application of an innovative system for storing grain under operating conditions. Agric Eng.

[33] Bergen GA, Jayas DS, White NDG (1993). Physical damage to peas and lentils due to free fall. Can Agric Eng.

[34] Perry JS, Hall CW (1966). Evaluating and reducing mechanical-handling damage to pea beans. Trans ASAE.

[35] Tang J, Sokhansanj S, Sosulski F (1991). Determination of the breakage susceptibility of lentil seed. Cereal Chem.

[36] Li X, Du Y, Guo J, Mao E (2020). Design, simulation, and test of a new threshing cylinder for high moisture content corn. Appl Sci.

[37] ASABE, Standards. S352. 2 moisture measurement—unground grain and seeds. American Society of Agricultural and Biological Engineers, Michigan; 2006

[38] Shahbazi F, Saffar A, Analooei M (2011). Mechanical damage to pinto beans as affected by moisture content and impact energy. Agric Eng Int CIGR J.

[39] Olisa BS, Awosanmi FE, Akinropo MS, Ishiak K, Danlami A, Okeke CU (2021). Differential response of commercial hybrid and open-pollinated maize seeds to mechanical damage during seed processing. Notulae Sci Biol.

[40] Evans MD, Holmes RG, McDonald MB (1990). Impact damage to soybean seed as affected by surface hardness and seed orientation. Trans ASAE.

[41] Khazaei J, Shahbazi F, Massah J, Nikravesh M, Kianmehr MH (2008). Evaluation and modeling of physical and physiological damage to wheat seeds under successive impact loadings: mathematical and neural networks modeling. Crop Sci.

[42] Stephens LE, Foster GH. Reducing damage to corn handled through gravity spouts. ASAE; 1976.

[43] Foster GH, Holman LE. Grain breakage caused by commercial handling methods. Agricultural Research Service, United States Department of Agriculture; 1973.

[44] Gatongi IN. Effects of mechanical injury upon corn (*Zea mays* L.) seed quality. M.S. Thesis, Mississippi State University. Mississippi State; 1982.

[45] Asiedu EA. Influence of delayed harvest, chilling and mechanical injury on quality of stored seed corn (*Zea mays* L.); 1986.

